# Towards Application of Electro-Fermentation for the Production of Value-Added Chemicals From Biomass Feedstocks

**DOI:** 10.3389/fchem.2021.805597

**Published:** 2022-01-19

**Authors:** Shohei Yamada, Yuki Takamatsu, Sota Ikeda, Atsushi Kouzuma, Kazuya Watanabe

**Affiliations:** Laboratory of Bioenergy Science and Technology, School of Life Sciences, Tokyo University of Pharmacy and Life Sciences, Hachioji, Japan

**Keywords:** bioelectrochemical systems, electrochemically active bacteria, electro-fermentation, extracellular electron transfer, redox balance

## Abstract

According to recent social demands for sustainable developments, the value of biomass as feedstocks for chemical industry is increasing. With the aid of metabolic engineering and genome editing, microbial fermentation has been developed for producing value-added chemicals from biomass feedstocks, while further improvements are desired for producing more diverse chemicals and increasing the production efficiency. The major intrinsic limitation in conventional fermentation technologies is associated with the need for balancing the net redox equivalents between substrates and products, resulting in limited repertories of fermentation products. One solution for this limitation would be “electro-fermentation (EF)” that utilizes bioelectrochemical systems for modifying the intracellular redox state of electrochemically active bacteria, thereby overcoming the redox constraint of fermentation. Recent studies have attempted the production of chemicals based on the concept of EF, while its utility has not been sufficiently demonstrated in terms of low production efficiencies. Here we discuss EF in terms of its concept, current status and future directions, which help us develop its practical applications to sustainable chemical industries.

## Introduction

The consumption of fossil resources continues to increase as the economy grows (Jones et al., 2021; Le Quéré et al., 2021). Fossil fuels have been the major primary energy sources for the human society, while the combustion of fossil fuels results in the emission of carbon dioxide, the largest cause of the global warming (Le Quéré et al., 2021). Nowadays, the global warming has started to impact climates and environments at many different places on our planet, resulting in rises in the sea level, local droughts, heavy rains and associated floods, and melting of frozen soils (Kerr, 2007). Such climate changes also affect natural ecosystems and agricultural/fishery production (Houghton, 2005; Kerr, 2007). For this reason, we should substantially replace primary energy sources from fossil fuels to other energy sources that less emit carbon dioxide.

Another concern on the use of fossil resources would be limited amounts of these resources deposited in our planet (Owen et al., 2010), despite that fossil resources, in particular petroleum, are the major feedstock of chemical industries for producing commodity chemicals essential for our daily life (Sheridan et al., 2021). One possible solution for this concern would be the use of renewable biomass as carbon feedstocks of chemical industries (Fagunwa and Olanbiwoninu, 2020). Biomass is renewable organic materials that come from plant and animals, and, since the use of biomass can be considered as “carbon neutral,” biomass-based industries are expected to contribute to the reduction in carbon-dioxide emission (Yang et al., 2017). In biomass-based industries, several chemicals, such as sugars, are used as primary feedstocks of microbial fermentation for producing value-added chemicals (Yang et al., 2017). This would be a relatively new application of microbial fermentation that has been closely related to our daily life from ancient times for producing foods (Ojha and Tiwari, 2016).

Microbial fermentation processes have commercially been used to produce some value-added chemicals in food and chemical industries (Yang et al., 2017). However, even though microbial metabolic pathways can be freely designed at the genome level using metabolic engineering and genome editing, fermentation processes are still capable of producing only limited species of chemicals (Gong et al., 2020). This notion is related to the major intrinsic limitation in conventional fermentation processes; namely, the need for balancing the net redox equivalents between substrates and products, resulting in limited repertories of fermentation products. One solution for this limitation would be “electro-fermentation (EF)” that utilizes bioelectrochemical systems (BESs) for modifying intracellular redox states of microbes, thereby overcoming the redox constraint of fermentation (Moscoviz et al., 2016; Schievano et al., 2016; Kracke et al., 2018). In EF, electrochemically active bacteria (EAB) that have extracellular electron transfer (EET) pathways for electrically interacting with extracellular conductive materials are considered particularly useful (Claassens et al., 2016; Logan et al., 2019). EF has been examined in laboratory for producing value-added chemicals (e.g., Flynn et al., 2010; Bursac et al., 2017), while further efforts are considered necessary for demonstrating its true power.

In this mini review, we first describe concepts of EF using EAB and subsequently summarize laboratory studies that have attempted to demonstrate the utility of EF. Based on these, we will discuss future directions of studies towards practical application of EF to sustainable chemical industries.

## Concepts of Electro-Fermentation

Fermentation is a catabolic process, in which organisms conserve energy by anaerobically decomposing organic matter. The human society has a long history of the utilization of microbial fermentation for food production and preservation (Bamforth and Cook, 2019), while it has also been expected that fermentation would be applicable to the production of commodity chemicals from renewable biomass feedstocks (Bastidas-Oyanedel et al., 2015). For this purpose, extensive studies have been performed, in which latest knowledge and technologies, such as metabolic engineering and genome editing, have been used for designing catabolic pathways at the genomic level. It is however still difficult for fermentation processes to produce many commodity chemicals at high yields, resulting in limited expectation in field of sustainable developments. The major cause for this difficulty is considered to be associated with “redox constraints” of microbial fermentation. In fermentation pathways, there exist oxidizing and reducing reactions, between which reducing equivalents are transferred via intracellular redox mediators, such as NADH and NADPH. Since amounts of these molecules in cells are limited, they must be efficiently recycled for fermentation processes to proceed (Kracke et al., 2018). In addition, total reducing equivalents must be balanced between substrates and products in order not to run short of oxidized or reduced form of these molecules in fermenting cells. This is the reason why fermentation processes are capable of producing limited species of chemicals, such as ethanol, lactate, succinate, and glutamate, at high yields (Kracke et al., 2018; Gong et al., 2020; Vassilev et al., 2021).

EABs are microbes that are capable of electrochemical interaction with electrode, and extensive studies have so far been conducted to elucidate how they interact with electrodes (Logan et al., 2019; Zhao et al., 2020). Studies have found that EAB have EET pathways that electrically connect intracellular redox reactions to extracellular redox materials (e.g., electrodes), so that electrons can efficiently be transferred across insulator cell membranes and walls (Logan et al., 2019; Zhao et al., 2020). To date, several different types of EET pathways have been uncovered, and these are broadly divided into two types, EET pathways in Gram-negative bacteria and those in Gram-positive bacteria. In Gram-negative bacteria, EET pathways extend over inner and outer membranes and are comprised of cytochromes as major electron-transfer components (Shi et al., 2016). In contrast, EET pathways in Gram-positive bacteria are designed to transfer flavins as electron carriers that facilitate electron transfer across thick cell walls (Light et al., 2018). Despite the above-mentioned differences, these EET pathways are conduits of electrons, facilitating electrons transfer in both directions (either from the inside to outside or from the outside to inside). For instance, the EET pathway of *Shewanella oneidensis* has a shallow gradient of redox potential (it gradually increases from the inside to outside), resulting in relatively high anodic current to high-potential electrodes compared to cathodic current from low-potential electrodes (Ikeda et al., 2021). These observations suggest that EET pathways can be used for controlling intracellular redox states (e.g., redox balances of NAD+/NADH) using extracellular electrodes whose potentials are arbitrarily set in BESs.

EF has been proposed based on the above-mentioned two ideas, namely the redox constraint of fermentation and the use of EET pathways for electron transfer across cell membranes. In EF, working electrodes in BESs are used to regulate intracellular redox states of EAB, thereby enabling redox-unbalanced carbon flows (Moscoviz et al., 2016). EF has therefore been expected to break through the limitation in conventional fermentation; namely, it can broaden product ranges and increase product yields. As also described above, both directions of electron transfer are possible, facilitating the development of anodic and cathodic EF processes. In an anodic EF, a high-potential working electrode serves as an electron acceptor, so that products can be more oxidized than substrates (Forster et al., 2017; Vassilev et al., 2021). In contrast, in a cathodic EF, a low-potential working electrode serves as an electron donor, so that products can be more reduced than substrates (Wu et al., 2019).

For EF producing value-added chemicals that are otherwise difficult to be produced at high yields, EAB whose catabolic pathways can be redesigned based on genomic information would be useful. To this end, EAB that are genetically accessible (whose catabolic pathways can readily be modified by genetic/genomic engineering) are desirable. One of such EAB is *S. oneidensis*; its genome has been sequenced, and catabolic pathways have been documented in detail (Hirose et al., 2019a). It is however unfortunate that the wild-type strain of *S. oneidensis* is incapable of glucose utilization (Hirose et al., 2019a), while a study has shown that introduction of two genes encoding sugar permease and hexose kinase in *Escherichia coli* facilitates this bacterium to grow on glucose (Nakagawa et al., 2015). In addition, since it has been known that expression of catabolic enzymes and electron-transport proteins in *S. oneidensis* is complexly regulated at the transcription level (Hirose et al., 2018), further studies on its regulatory mechanisms would be necessary for EF to efficiently utilize designed catabolic pathways (Ikeda et al., 2021). Another EAB that is genetically accessible is *Geobacter sulfurreducens*; the use of this bacterium in BESs is attractive in terms of its high electrochemical activity (Ueki, 2021), while genetic manipulation of this bacterium would be more difficult than *S. oneidensis* owing to its inability to grow aerobically. *Acidithiobacillus ferrooxidans* is also an EAB that has been suggested to be useful in biotechnology (Kernan et al., 2016), while its utility would be related to microbial electrosynthesis (Wood et al., 2021) rather than EF in terms of its ability for carbon and nitrogen fixation. We however consider that this bacterium is also attractive for EF, since its growth under extremely acidic conditions (optimum at pH 2) facilitates efficient proton uptake that is always associated with electron uptake from cathodes for producing reduced chemicals. We therefore wait for the development of efficient genetic manipulation techniques for this bacterium.

In summary, EF is physiological means for controlling carbon flows in EAB. EF would be particularly useful for producing value-added chemicals, if EF is combined with metabolic engineering of catabolic pathways based on genomic information.

## Laboratory Demonstrations of Electro-Fermentation

Several studies have been conducted for demonstrating the utility of EF. Herein we list up these studies (Table 1) and discuss the current status of EF. One of such studies has been reported by Forster et al. (2017), in which they used an *E. coli* strain expressing the EET pathway of *S. oneidensis* for producing acetoin from glucose. This is an example of anodic EF, in which excess reducing power generated in a catabolic pathway to produce acetoin from glucose was discharged to an anode, resulting in 3.6-fold increase in the production titer compared to that without electron discharge (Forster et al., 2017). An EET pathway-expressing *E. coli* strain was also used in cathodic EF for producing succinate from glucose (Wu et al., 2019); the engineered strain was cultivated in the presence of a low-potential electrode, resulting in the production of 30.5 mM succinate at a molar yield of 1.1. In these studies, however, redox mediators, such as methylene blue and neutral red, were added for gaining positive effects of extracellular electrodes, and it is likely that the introduced EET pathways did not effectively operate in *E. coli*. This notion is supported by the fact that the engineered *E. coli* strain generated anodic current at 10 µA cm^−2^ (Forster et al., 2017), less than 1/10 compared to that generated by *S. oneidensis* (Kasai et al., 2019). Besides, since mediator compounds are unstable and expensive, mediator-less EF is favorable for industrial application.

**TABLE 1:** Studies examining the utility of EF for the production of value-added chemicals.

EF type	Organism	Substrate	Product	Genetic manipulation	Electrode potential (V vs. SHE)^a^	Redox mediator	Efficiency^b^	References
Anodic	*Escherichia coli*	Glucose	Acetoin	Yes	+0.2	Yes	0.79	Forster et al. (2017)
	*Escherichia coli*	Glycerol	Ethanol	Yes	+0.2	Yes	12 mg h^−1^, 0.35, 55 g L^−1^	Sturm-Richter et al. (2015)
	*Escherichia coli*	Lactate	Ethanol	Yes	+0.4	No	0.08 mg h^−1^, 10 g L^−1^	TerAvest et al. (2014)
	*Shewanella oneidensis*	Glycerol	Ethanol	Yes	+0.4	No	0.84, 1.3 g L^−1^	Flynn et al. (2010)
	*Shewanella oneidensis*	Lactate	Acetoin	Yes	0	No	0.91 mg h^−1^, 0.52, 0.24 g L^−1^	Bursac et al. (2017)
Cathodic	*Escherichia coli*	Glucose	Succinate	Yes	−0.45	Yes	0.55	Wu et al. (2019)
	*Shewanella oneidensis*	Acetoin	2,3-Butanediol	Yes	−0.03	No	2.7 mg L^−1^	Tefft and TerAvest (2019)
	*Geobacter sulfurreducens*	CO_2_, Succinate	Glycerol	No	−0.4	No	0.8 g L^−1^	Soussan et al. (2013)
	*Clostridium pasteurianum*	Glycerol	1,3-Propanediol	No	−0.4	Yes	0.41	Utesch et al. (2019)

a SHE, standard hydrogen electrode.

b Rates (mg L^−1^), yields (no unit) and/or titers (g L^−1^) are presented.

The use of innate EAB, such as *S. oneidensis*, in EF has also been examined. It has been shown that anodic EF using a metabolically engineered *S. oneidensis* strain produced ethanol from glycerol at a molar yield of 0.84, which was higher than that of EF using an engineered *E. coli* strain (0.53) (Flynn et al., 2010). Other studies have also attempted the use of engineered *S. oneidensis* strains in EF, and these include anodic EF for producing acetoin from lactate (Bursac et al., 2017) and cathodic EF for producing 2,3-butanediol from acetoin (Tefft and TerAvest, 2019). As summarized in Table 1, however, despite that some merits of EF can be observed in these studies, efficiencies (e.g., titers, yields and rates) for the production of chemicals in these EF processes are not high compared to those reported for conventional fermentation processes. Further efforts are therefore necessary to increase the efficiency of EF for producing value-added chemicals.

## Towards Practical Application of Electro-Fermentation

For practical application of EF, researchers should focus their studies on developing methodologies to increase production efficiencies in EF. For instance, succinate production by the EET-expressing *E. coli* strain is ∼25 times slower than those achieved by engineered *E. coli* strains grown under conventional fermentation conditions (Cao et al., 2013). Several different approaches would be possible for increasing production efficiencies of EF, and these include the development of efficient BES reactors and the improvement of electrochemical activities of EAB. In terms of BESs, high-performance electrodes that have been developed for microbial fuel cells (e.g., Bian et al., 2020) would be applicable to EF. These electrodes include carbon nanotube-coated graphite felt (Zhao et al., 2011) and carbon nanotube-modified reticulated vitreous carbon (Jourdin et al., 2014). These electrode materials have large surface areas for enhancing bacterial cell attachment and macroporous structures for efficient substrate diffusion, thereby increasing current densities in BESs. In addition, insertion of multiple electrodes into BESs would also contribute to increases in numbers of EAB cells accessible to electrodes, facilitating current-associated bioproduction per reactor volume (Shimoyama et al., 2008).

On the other hand, genetic-engineering approaches have been examined for increasing electrochemical activities of EAB, and the use of engineered EAB would be feasible approaches for increasing efficiencies of EF. These studies are associated with the identification of cellular functions that affect electrochemical activities of EAB, and these include EET, transcriptional regulation, and biofilm formation (Delgado et al., 2019; Kasai et al., 2019; Vellingiri et al., 2019; Cheng et al., 2020). To cite an instance, Kasai et al. (2019) have reported that an engineered strain of *S. oneidensis* overexpressing an adenylate cyclase gene generated 2.2-times higher anodic current than the wild-type strain. In this engineered strain, the intracellular concentration of cyclic AMP, a key signaling molecule regulating the expression of EET and catabolic pathways in *S. oneidensis*, was significantly elevated, resulting in the coordinated upregulation of these functions. In addition, Cheng et al. (2020) have reported that expression of an exogenous adenylate cyclase gene in *S. oneidensis* resulted in increased cathodic current in the presence of fumarate as the electron acceptor. Another approach is the engineering of cell-surface structures involved in the attachment of bacterial cells to electrodes and biofilm formation (Kouzuma et al., 2010). For instance, Kouzuma et al. (2010) have reported that the disruption of a gene involved in cell-surface polysaccharide synthesis in *S. oneidensis* facilitates attachment to electrodes, thereby enhancing anodic current. Studies have also shown that cyclic diguanosine monophosphate (c-di-GMP) serves as an intracellular signaling molecule involved in biofilm formation on electrodes by *S. oneidensis* (Matsumoto et al., 2021), and the overexpression of a diguanylate cyclase gene increases intracellular c-di-GMP concentration, resulting in enhanced biofilm formation and current generation (Liu et al., 2015). Taken together, the above-mentioned studies demonstrate that genetic engineering is a possible approach for improving electrochemical activities of EAB, thereby enhancing performances of EF. In order to further increase their electrochemical activities, future studies will examine the combination of multiple engineering strategies in one EAB strain. In addition, electrogenetics, a method to control gene expression using electrodes (Hirose et al., 2019b), would be useful for coordinated expression of genes in EAB engineered for EF.

## Discussion

**FIGURE 1:**
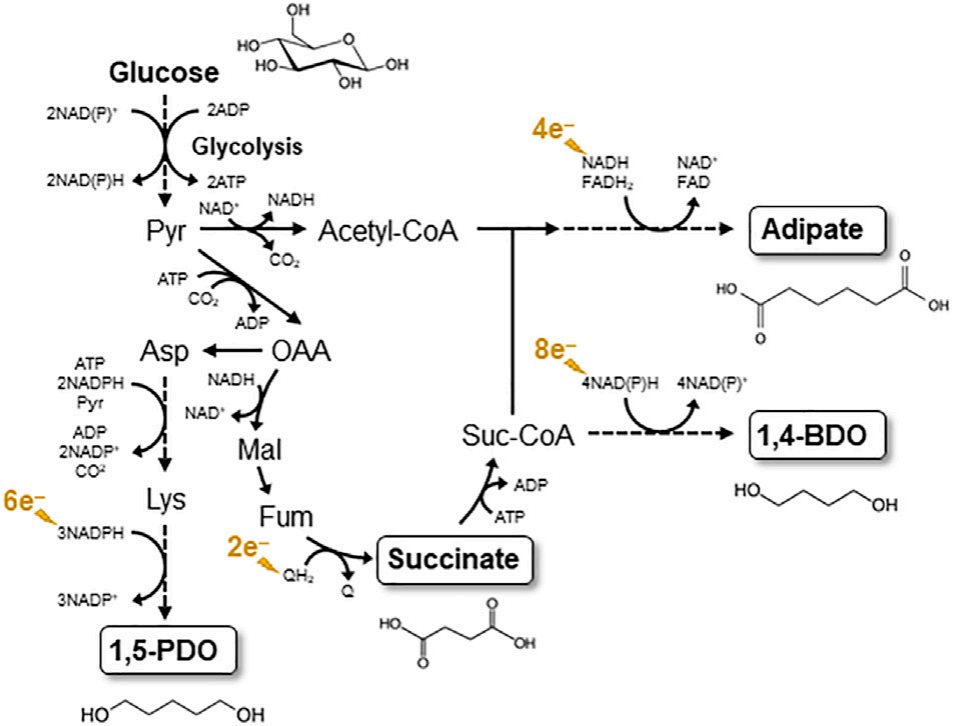
Biosynthesis pathways for C4 and C5 diols and dicarboxylic acids considered as target products of EF. The biosynthesis pathways are depicted based on reports by Yim et al. (2011), Zhao et al. (2018), and Cen et al. (2021). Pyr, pyruvate; Mal, malate; Fum, fumarte; Q, oxidized quinine; QH2, reduced quinone; Suc-CoA, succinyl-CoA.

Here we briefly discuss target compounds of EF, with which the utility of EF can effectively be shown. Among the two possible EF processes (anodic and cathodic), production of reduced compounds by cathodic EF would be attractive, since electricity is a cheap reducing power compared to biomass-derived feedstocks, such as glucose. One of such EF targets would be diols and related compounds that serve as building blocks for commodity polymers, such as urethane. For instance, succinate and 1,4-butanediol (1,4-BDO) have been produced from glucose at the commercial level in conventional fermentation processes (Yim et al., 2011; Burgard et al., 2016; Meng et al., 2016), in which EF is considered useful for the production of 1,4-BDO from succinate (Figure 1). This is because, as shown in Figure 1, much reducing power is necessary for the conversion, and the expense of organic feedstocks for supplying the reducing power is not economically desirable. Since diols (e.g., 1,4-BDO) are important in chemical industries, we suggest the need for efficient and inexpensive production of diols from biomass feedstocks, and bioprocesses incorporating EF would be suited for this demand. Similarly, we expect that adipate and 1,5-pentanediol, also building blocks of polymers, would beneficially be produced by EF (Figure 1).

EF is attractive means for physiologically controlling EAB for producing value-added chemicals from biomass feedstocks, for which efficient electron transfer between intracellular catabolic pathways and extracellular electrodes via EET pathways is needed. In order for EF to move forward towards practical applications, further work would be necessary for increasing their production efficiencies, for which understanding regulatory mechanisms for catabolic and electrochemical activities of EAB would be important. It is also important for EF to target compounds of industrial and economic interests that are otherwise difficult to be produced efficiently (such as, diols). We expect that EF will be accepted as an attractive option in sustainable bioindustry that is particularly useful for producing reduced chemicals.
